# Fuzzy species revisited

**DOI:** 10.1186/1741-7007-11-41

**Published:** 2013-04-15

**Authors:** William P Hanage

**Affiliations:** 1 Center for Communicable Disease Dynamics, Harvard School of Public Health, Boston, MA 02115, USA

## 

How species should be defined for bacteria, and the debate over whether such things exist in a form worthy of the name, have been long and mostly sterile controversies. There are several reasons for this, including the difficulty in applying concepts developed for one kingdom of life to another, but in recent years the importance of horizontal gene transfer or recombination has also become a point of contention. Few now disagree that the movement of genetic material between lineages is an essential source of evolutionary innovation, and that bacteria tend towards a 'plug and play' strategy that allows genes specific for quite narrow segments of niche space to be shared among multiple species. (I am using the word 'species' in the sense of 'a group of organisms given that name' and do not intend this to be a comment on whether any 'species concepts' are viable.) However, homologous recombination is also more than capable of transferring core housekeeping loci between species [1].

In our 2005 paper [2] we suggested the term 'fuzzy species' for those that did not form clear and distinct sequence clusters, as assayed by phylogenies of concatenated housekeeping genes. This was illustrated with the example of the *Neisseria*. Using thousands of sequences gleaned from molecular epidemiology we showed that the sister 'species' *Neisseria meningitidis *and *Neisseria lactamica *were not perfectly distinguished by the sequences of seven housekeeping genes, and that there was good evidence for severe taxonomic confusion among other named *Neisseria *species. In contrast, the gonococcus (*Neisseria gonorrhoeae*) was clearly distinct. This is likely due to ecology. The imperfect separation we observed between *N. lactamica *and *N. meningitidis *was due to mosaic genotypes containing sequence 'typical' of both species. These organisms both colonize the human nasopharynx and so are likely to have frequent opportunities for recombination. In contrast the gonococcus colonizes a different mucosal surface (at least most of the time [3]) and this means the opportunities for recombination with other *Neisseria *are limited.

Such 'fuzziness' has gone on to be found among other recombining bacteria [4,5] and indeed Archaea [6]. However, the significance of this observation can be overstated. It does not mean that bacterial species definitions are all inherently insecure, nor even that this is the case for those species where fuzziness is observed. We must distinguish between species definitions and species concepts. A definition is merely the criteria used to classify an organism, and is important for practical reasons. A coherent species concept that can be applied throughout the kingdoms of life is still elusive. However, if we are agnostic about whether 'species' exist in a way that can be justified by philosophers, we can still ask whether clusters of related strains exist, what the characteristics of those clusters might be, and whether this can be helpful for classification. This is the approach used by multi-locus sequence analysis (MLSA). In some cases, researchers have made use of the internet to allow scientists throughout the academic community to contribute to the study of these 'species' clusters [7]. Moreover, it has been possible to model the emergence of such clusters, and examine the role of recombination in generating them [8,9].

This work has suggested that bacteria may fall into 'clonal' and 'sexual' species, with the latter distinguished from the former by higher recombination rates. In 'clonal' species, clusters are generated in the main by mutational processes and are predicted to appear under neutrality through the random birth and extinction of lineages. In a 'sexual' species, the observed cluster is the result of recombination between different members of the species cluster preventing the budding of distinct daughter lineages. This theory predicts that limiting recombination can lead to a single cluster separating in two, in a way that bears comparison with reproductive isolation. However, removal of the barrier to recombination will result in the two clusters merging once more, unless sufficient divergence has occurred [10]. It has even been suggested that there may be evidence for such 'despeciation' occurring in nature [11].

## Critiques of fuzzy species

While homologous recombination between species seems quite common, describing the structure and characteristics of species clusters in these terms has attracted criticisms, and these deserve attention. We must wonder whether such 'intermediate' forms as we find in fuzzy species are the consequence of mixed cultures. Even the best microbiological technique will occasionally produce such errors. When we isolate DNA from such mixtures and sequence it, the result could be the appearance of a mosaic genotype (see [12] for an example). The more samples you sequence, the greater the chance you will find one of those rare errors. Furthermore, even assuming that the 'intermediate forms' are not errors, are they of any biological significance? Mosaic genotypes are infrequent, and instead of reflecting how common interspecies recombination is, they could be interpreted as showing how rare it is, to produce such limited evidence of hybridization.

There is ample reason to discount the suggestion that all fuzziness is the result of laboratory error. Were this to be the case, mosaic genotypes should be distributed randomly around the 'species' cluster, rather than clustering into specific lineages that seem to be more likely to acquire 'foreign' DNA. However, in the case of *N. meningitidis*, a population genetic analysis of thousands of genotypes shows clear evidence for groups of closely related isolates that appear likely to have picked up *N. lactamica *DNA on independent occasions, and are not simply defined as similar on account of their partially *N. lactamica *ancestry [13]. It is reasonable to suggest that such mosaicism may be due to some biological feature of those isolates that means they have been more prone to recombination with the sister species in the past.

A better critique is that the discussion of fuzzy species has made use of a tiny fraction of the core genome. We increasingly enjoy access to genomic data, and it seems improbable at this point that this will reveal any 'intermediates' between named species. Instead sequencing more loci should result in improved definition and resolution of the species cluster in question.

## The potential of population genomics

Population genomic studies are increasingly revealing substructure within species clusters. In many cases what we think of as bacterial species are composed of very many distinct lineages that recombine with one another often enough to spread genetic innovation, and are grouped together by crude phylogenetic approaches. The differences between such subclusters are often not trivial. The consequences, for example, of acquiring toxin genes are well appreciated, and the gain and loss of mobile elements may be very rapid in evolutionary terms [14]. More subtle distinctions have also been reported: for example, it has been known for some time that some 'atypical' isolates of *Streptococcus pneumoniae *that lack the major polysaccharide antigen have a specific tendency to cause conjunctivitis (see [15] for a well documented outbreak). These organisms were of a single closely related lineage by conventional genetic typing methods, but not obviously divergent from the rest of the named species [16]. However, analysis of the whole genome shows that the conjunctivitis-associated lineage is divergent from the rest of the species [17], and probably harbors its own distinct set of accessory loci. In genomic terms, these organisms are clearly distinct from *S. pneumoniae *and they may deserve their own species name.

The debate has hence moved on to the consequences of the recombination that produces the 'fuzziness' we observe in clusters we identify by MLSA, and how that relates to the ecology of the organism [18]. In some cases, recombination seems to occur at a rate sufficient to uncouple a selected locus from the rest of the genome [19]. This suggests the potential for a gene-centered theory of ecology, which addresses the 'niches' (including genomic background) in which a gene will thrive. Genomic data are ideal for such questions, and are increasingly available for hundreds or thousands of isolates. The clusters of genomes we observe reflect the niche structure on which the organism has evolved, which will also be related to the presence of accessory loci, and the opportunities for recombination between lineages. It may be that the fuzzy species we observe are best understood as the inevitable consequence of fuzzy niches.

## Note

This article is part of the *BMC Biology *tenth anniversary series. Other articles in this series can be found at http://www.biomedcentral.com/bmcbiol/series/tenthanniversary.
